# Assessing the impact of social determinants of health on diabetes severity and management

**DOI:** 10.1093/jamiaopen/ooae107

**Published:** 2024-10-25

**Authors:** Xiyu Ding, Hadi Kharrazi, Akihiko Nishimura

**Affiliations:** Biomedical Informatics and Data Science, Division of General Internal Medicine, Johns Hopkins School of Medicine, Baltimore, MD 21205, United States; Biomedical Informatics and Data Science, Division of General Internal Medicine, Johns Hopkins School of Medicine, Baltimore, MD 21205, United States; Department of Health Policy and Management, Johns Hopkins Bloomberg School of Public Health, Baltimore, MD 21205, United States; Department of Biostatistics, Johns Hopkins Bloomberg School of Public Health, Baltimore, MD 21205-2179, United States

**Keywords:** social determinants of health, type 2 diabetes mellitus, HbA1c, self-controlled study design

## Abstract

**Objective:**

Adverse Social Determinants of Health (SDoH) are considered major obstacles to effective management of type-2 diabetes. This study aims to quantify the impact of SDoH factors on diabetes management outcomes.

**Materials and Methods:**

We quantified the joint impact of multiple SDoH by applying a self-control case series method—which accounts for confounding by using individuals as their own control—to electronic health record data from an academic health system in Maryland.

**Results:**

We found a consistent increase in HbA1c levels associated with SDoH across alternative study designs. The estimated total contributions of SDoH ranged 0.014–0.065 across the alternative designs. Transportation issues demonstrated particularly significant contributions, with estimates of 0.077–0.144. When assuming SDoH’s risk window to be ±45 days, for example, the total contribution was estimated to be 0.065 (95% CI [0.010, 0.120]) increase in HbA1c and the transportation issues’ contribution 0.134 (95% CI [0.020, 0.249]).

**Discussion and Conclusion:**

Our result suggests that reducing transportation barriers may be an effective SDoH intervention strategy for diabetes management; however, the clinical impact of such interventions warrants further investigation.

## Introduction

Diabetes is the eighth leading cause of death in the United States,^1^ with 14.7% of all United States adults (37.1 million) living with the disease.^2^ In 2017, the American Diabetes Association reported an estimated total cost of diagnosed diabetes to be $327 billion, contributing up to 25% of total United States healthcare costs.^3^ Effective management of diabetes by maintaining blood glucose levels can prevent many of its clinical and economic consequences. However, a significant proportion of diabetes patients failed to achieve the recommended target for such glycemic control.^4^

Past studies have shown that diabetes disproportionately affects racial/ethnic minorities and socioeconomically disadvantaged populations.^5–8^ Social Determinants of Health (SDoH) have been recognized as major drivers of health inequities in diabetes risks and outcomes.^9^^,^^10^ Understanding the impact of SDoH on the trajectory of patients with diabetes is, therefore, essential for developing effective intervention strategies and mitigating the disease’s adverse consequences.

Relationships between diabetes outcomes and SDoH factors have previously been investigated. Most studies, however, consider individual SDoH factors in isolation without accounting for the fact that they often occur in combination.^10–16^ Understanding their relative important is important for policymakers when developing targeted interventions to address specific SDoH factors and allocating resources more effectively.^17^

Our study quantifies the impact of SDoH on Type 2 Diabetes Mellitus (T2DM) management by using electronic health record (EHR) data from an academic health system in Maryland. Previous work in this area has relied on case-control design, comparing the disease outcomes of patients who had social issues with those who did not.^13^ However, such an approach has major shortcomings. First, the absence of SDoH issues cannot be ascertained from EHR data; an absence of diagnosis codes only means that a clinician did not report the issues, not that the patients had not experienced them.^18^ Second, even if we could ascertain the absence of SDoH issues, using a case-control design to draw a causal conclusion is known to be prone to residual confounding when using EHR data.^19^ To avoid these potential pitfalls, we used a self-control case series (SCCS) method to examine the impacts of adverse SDoH on T2DM management, taking individuals as their own controls.^20^^,^^21^ By only including individuals who experienced adverse SDoH effects, this method allowed us to quantify the SDoH effects with all time-invariant confounding factors eliminated.

## Methods

### Cohort and study period

Our study population included 137 366 patients with T2DM who were 16 years or older on January 1st, 2019, and received care at the Johns Hopkins Health System facilities from 2017 to 2019.^22^ We identified patients with diabetes using the SUPREME-DM phenotype criteria based on diagnosis codes, lab results (Hemoglobin A1c ≥6.5%, Fasting Plasma Glucose ≥126 mg/dL or Oral Glucose Tolerance ≥200 mg/dL), or orders of medication indicated only for type-2 diabetes.^23^ The initial study population included 139 251 patients. We subsequently removed 1885 patients with missing sex or race, or a reported negative age, during the study period.

### Exposure: social determinants of health

We used a list of ICD-10-CM codes (Table S1) to identify social risk factors that may affect a patient’s T2DM management in the following domains: (1) financial resource strain (finance), (2) food deficiency (food), (3) residential instability (housing), (4) transportation issues (transportation), (5) health care access, (6) social connections and isolation (isolation), and (7) stress. This ICD-10 list was adapted from the compendium of SDoH codes developed by the Social Interventions Research and Evaluation Network (SIREN).^24^

**Figure 1.**
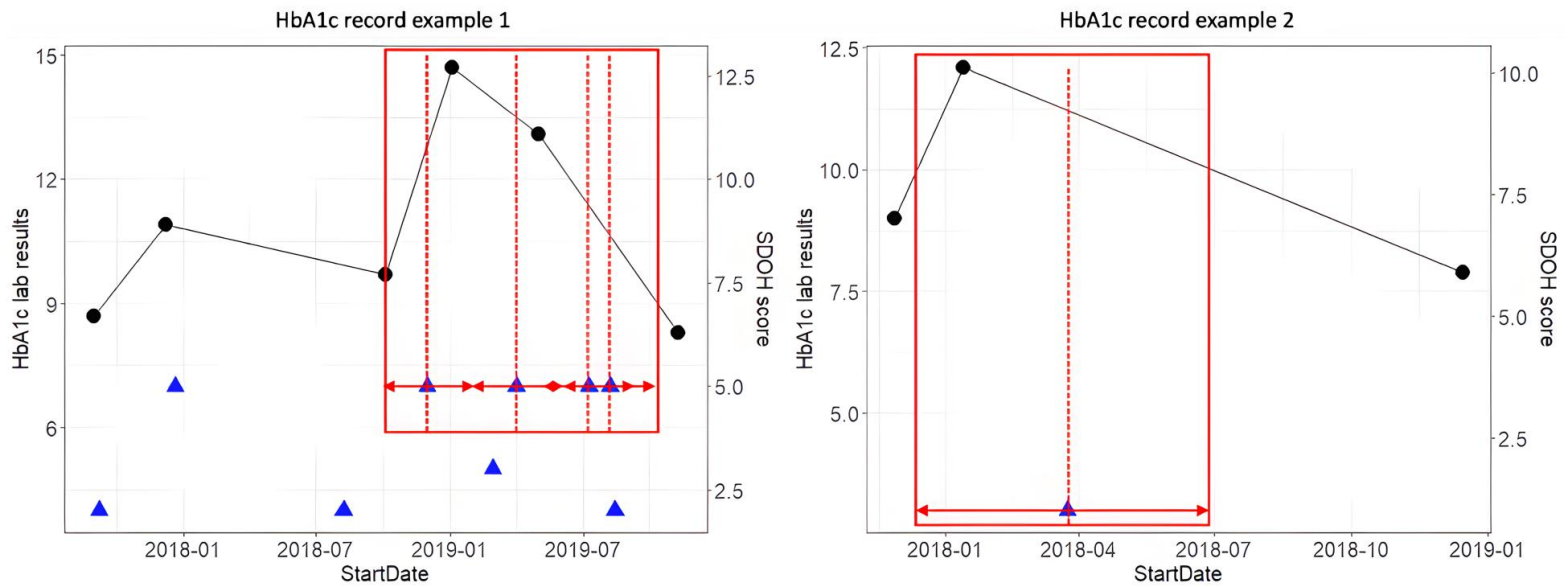
An example of HbA1c records. Both patients in the example were experiencing at least one social issue. Round black dots: patients’ HbA1c levels; triangle blue dots: patients’ SDoH scores (the number of social issues the patient experienced); the red square: defined risk periods of the SDoH issues.

A record of adverse SDoH in a patient’s EHR confirms the SDoH experience at a specific time point; however, we cannot determine based on this record when the adverse SDoH began or was resolved. To address this difficulty in determining the exact time span of a patient experiencing social issues, we considered multiple definitions of the SDoH exposures, corresponding to risk windows of 30, 45, 60, 90, 120, or 180 days before the first and after the last ICD-10-CM diagnosis (Figure 1). We considered the patient to be under constant and consistent influence of an SDoH factor during the risk window.

### Outcome: HbA1c as indicators of type 2 diabetes severity

We used lab-obtained Hemoglobin A1c (HbA1c) level as a measure of diabetes severity. HbA1c measures the percentage of hemoglobin protein in the blood that is glycated.^25^ HbA1c reflects a patient’s average blood glucose level for the last 2-3 months and how well diabetes has been controlled.^25^ Patients with at least two HbA1c test results, one impacted by the SDoH factors and another unimpacted, were included in the data analyses.

### Statistical analysis

We applied the continuous self-control case series (CSCCS) model,^20^^,^^21^ to quantify the effects of social risk factors on diabetes severity and management (HbA1c). The CSCCS model assumes the following relationship between the outcome and predictors:

$$y_{ij}|X_{ij}=\alpha_{i}+\beta^{T}X_{ij}+\epsilon_{ij},\;\epsilon_{ij}∼N\left(0,\sigma^{2}\right).$$

In our case, $y_{ij}$ represents the *i*th patient’s HbA1c value measured at the *j*th visit; $X_{ij}$ the SDoH exposure status; β the coefficients measuring the effects of SDoH factors on the response $y_{ij}$; $\alpha_{i}$ the patient-specific baseline HbA1c level.

We conducted one analysis in which we included all the individual SDoH factors as separate predictors, and another analysis in which we combined the individual factors into a single overall indicator. We calculated *P*-values to assess the significance of associations and reported confidence intervals at 95% levels. All statistical analyses were conducted in R 4.2.2.

## Results

### SDoH frequency

Out of the 137 366 patients with diabetes, we identified 10 707 (7.79%) individuals with records of SDoH issues from 2017 to 2019. Specifically, 716 (0.52%) patients experienced financial issues, 461 (0.33%) food deficiency, 2250 (1.64%) housing instability, 2946 (2.14%) issues with transportation, 3713 (2.70%) issues with health care access, 5140 (3.74%) stress, and 4621 (3.36%) issues with social connections and isolation.

### Patient characteristics

**Figure 2.**
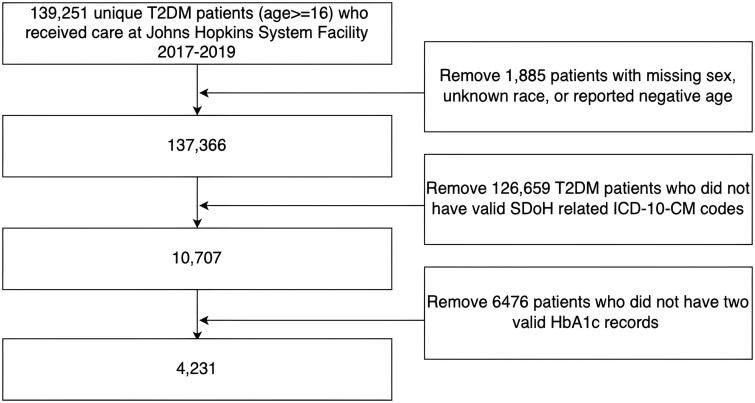
CONSORT (Consolidated Standards of Reporting Trials) diagram depicting the study population selection process.

For the CSCCS analyses, the study cohort consisted of 4231 patients with at least two HbA1c test results along with a record of SDoH (Figure 2). The mean age was 61.4 (SD = 13.8). Women comprised 54.9%. The racial composition was 47.1% White, 42.9% Black, 4.4% Asian, and 5.7% Others. The mean adaptive Diabetes Complications Severity Index (aDCSI) was 2.05 (SD = 2.16) (Table 1).^26^

**Table 1.** Patient characteristics by SDoH exposure (*N* = 4231).

SDoH^a^	Finance	Food	Housing	Transportation	Health care access	Stress	Isolation	Overall
	(*N* = 280)	(*N* = 188)	(*N* = 774)	(*N* = 1279)	(*N* = 1454)	(*N* = 2112)	(*N* = 1849)	(*N* = 4231)
**Gender**								
Female	164 (58.6%)	109 (58.0%)	347 (44.8%)	682 (53.3%)	794 (54.6%)	1230 (58.2%)	1035 (56.0%)	2323 (54.9%)
Male	116 (41.4%)	79 (42.0%)	427 (55.2%)	597 (46.7%)	660 (45.4%)	882 (41.8%)	814 (44.0%)	1908 (45.1%)
**Age**								
Mean (SD)^c^	61.4 (11.7)	61.3 (14.3)	56.8 (11.6)	60.8 (13.6)	62.9 (13.3)	62.4 (13.6)	62.8 (14.4)	61.4 (13.8)
Median [Min, Max]	62.5 [26.3, 88.0]	63.4 [21.4, 89.5]	57.1 [18.8, 88.0]	61.4 [18.1, 89.5]	64.1 [16.0, 89.5]	63.4 [16.0, 89.5]	64.0 [16.0, 89.5]	62.0 [16.0, 89.5]
**Race**								
Asian	3 (1.1%)	10 (5.3%)	5 (0.6%)	95 (7.4%)	46 (3.2%)	71 (3.4%)	57 (3.1%)	186 (4.4%)
Black	151 (53.9%)	80 (42.6%)	436 (56.3%)	536 (41.9%)	622 (42.8%)	882 (41.8%)	745 (40.3%)	1813 (42.9%)
Other	26 (9.3%)	8 (4.3%)	51 (6.6%)	84 (6.6%)	97 (6.7%)	114 (5.4%)	105 (5.7%)	240 (5.7%)
White	100 (35.7%)	90 (47.9%)	282 (36.4%)	564 (44.1%)	689 (47.4%)	1045 (49.5%)	942 (50.9%)	1992 (47.1%)
**Ethnicity**								
Non-Hispanic	255 (91.1%)	181 (96.3%)	738 (95.3%)	1205 (94.2%)	1378 (94.8%)	2031 (96.2%)	1777 (96.1%)	4052 (95.8%)
Hispanic	25 (8.9%)	7 (3.7%)	36 (4.7%)	74 (5.8%)	76 (5.2%)	81 (3.8%)	72 (3.9%)	179 (4.2%)
**aDCSI** ^b^								
Mean (SD)	2.70 (2.36)	2.69 (2.48)	2.58 (2.25)	1.60 (1.95)	2.46 (2.27)	2.16 (2.19)	2.42 (2.25)	2.05 (2.16)
Median [Min, Max]	2.00 [0, 9.00]	2.00 [0, 9.00]	2.00 [0, 10.0]	1.00 [0, 9.00]	2.00 [0, 10.0]	2.00 [0, 10.0]	2.00 [0, 10.0]	2.00 [0, 10.0]

a SDoH: social determinants of health

b aDCSI: adaptive Diabetes Complications Severity Index

c SD: standard deviation

### CSCCS analyses results

**Figure 3.**
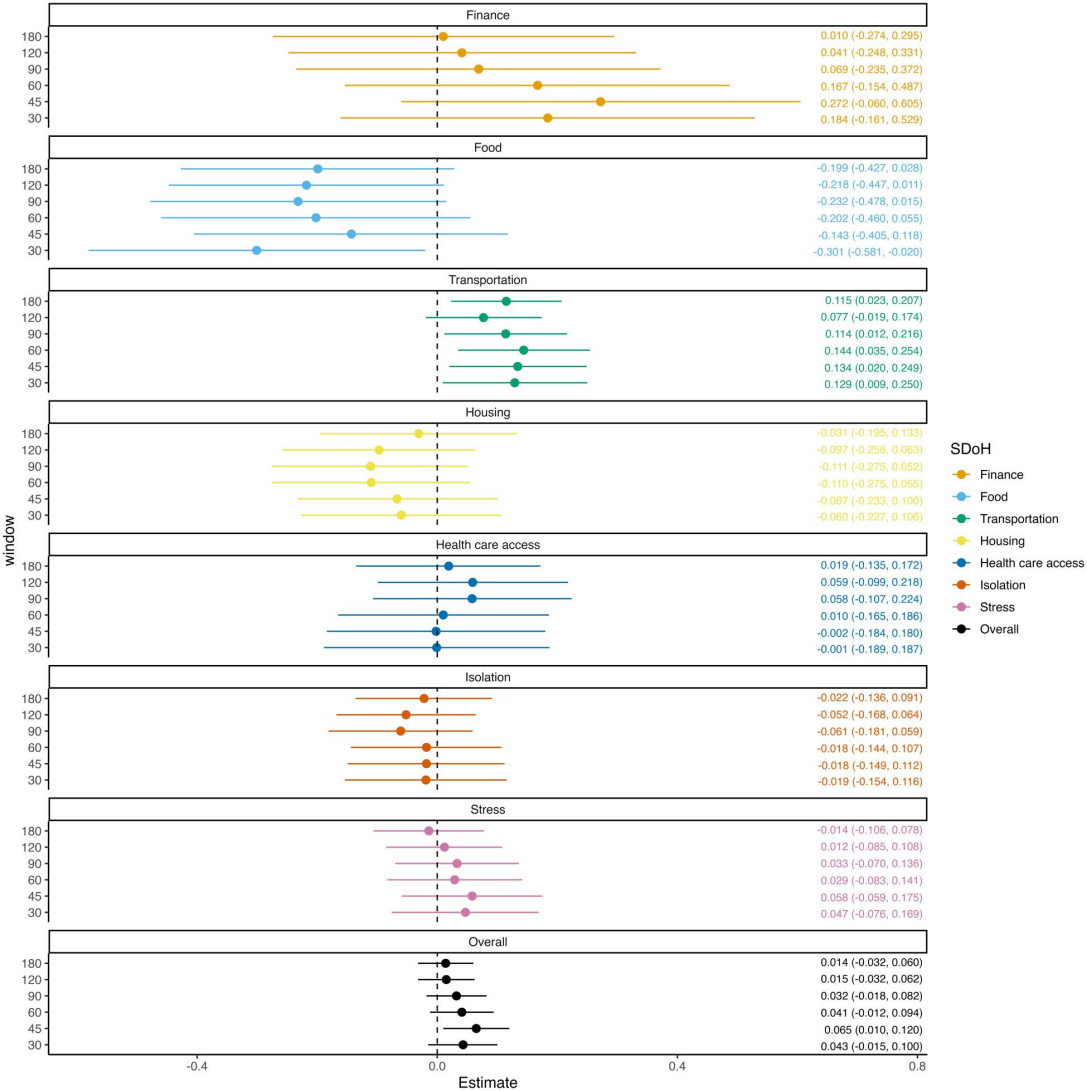
Forest plot showing the results of the CSCCS model estimating the impact of SDoH on HbA1c evaluated at different risk window periods.

Figure 3 summarizes the effects of SDoH factors on the HbA1c level estimated under the CSCCS model. The figure presents the estimates and 95% CIs across the different risk window periods to assess the robustness of the results. Overall, experiencing social issues was associated with an increase of HbA1c level (%) by 0.065 (95% CI [0.010, 0.120]) evaluated at a window period of ±45 days. As for individual SDoH factors, having transportation issues was associated with an increase of HbA1c by 0.115 (95% CI [0.023, 0.207]), 0.114 (95% CI [0.012, 0.216]), 0.144 (95% CI [0.035, 0.254]), 0.134 (95% CI [0.020, 0.249]), 0.129 (95% CI [0.009, 0.250]) evaluated at window periods of ±180, 90, 60, 45, 30 days. Other SDoH factors were not found to have statistically significant associations with changes in the HbA1c levels across multiple window sizes.

## Discussion

Social challenges have been found to play a significant role in T2DM management.^10^^,^^13^^,^^27^^,^^28^ In our study, we extracted SDoH from an academic health system EHR and applied the CSCCS model to assess the impacts of SDoH on HbA1c. Our analysis demonstrates that SDoH explains a statistically significant increase in HbA1c, although clinical significance should be further investigated. Our analysis of individual SDoH components further identifies the transportation barrier to be a potentially influential contributor to the increase in HbA1c. Our findings reinforce previous studies also suggesting travel burdens as a potential barrier to glycemic control.^29–31^

While our estimate indicates only a modest increase, the estimate is likely biased towards the null due to under-reporting of SDoH in EHR and their actual impacts are likely greater. A previous study by Chambers et al,^31^ in which social needs were collected through survey, found that 10.1% of their patients experienced housing issues, 7.1% food insecurity, and 6.9% transportation issues. On the other hand, the confirmed prevalence rates of SDoH are much lower in our study. In particular, we found only 2.1% of our patients have transportation issues recorded, compared to their 6.9%. This suggests that we were able to identify only one-third of the actual cases and, in turn, that our effect sizes have been diluted by one-third. In other words, the true impact of transportation issues could be 0.3% increase in HbA1c, instead of 0.1% we found. This 0.3% increase is enough to significantly increase the risk of developing composite vascular events, especially among patients with HbA1c around the 6.5% diagnostic cutoff line.^31^^,^^32^ Also worth noting is that the estimated effect only represents the population average and, in practice, some groups are likely more disproportionately affected by SDoH.

Potential underreporting of SDoH also explains why other factors, such as housing, were found to be significantly related to poor HbA1c control in other studies but not ours.^10^^,^^31^ Another study by Kepper et al^33^ also reported that housing and economic circumstances, two of the most commonly recognized social needs in the provider interviews, were rarely documented with ICD-10 codes. So far, capturing SDoH from EHR systems is very limited by the lack of standardized data elements, limited interoperability, and workflow and time constraints of healthcare providers.^34^ To better quantify the impact of SDoH on diabetes management, we need to improve the recording and collection of social issues in EHR systems.

We also note the importance of taking into consideration contextual differences in the SDoH definitions when interpretating their effects. Our study found food deficiency to be associated with a decreased HbA1c level when evaluated at the window period of ±30 days. This can be explained by the fact that patients with food deficiency likely have lower energy intake and therefore lower HbA1c. Previous studies found food insecurity, as opposed to food deficiency, is associated with higher HbA1c potentially due to consumption of low-cost and highly processed food that is high in refined carbohydrates and fats instead of nutrient-rich food.^35^^,^^36^ More generally, different aspects of the same SDoH factor, such as food deficiency versus food insecurity, can produce different effects on T2DM management.

This study has several limitations. First, our construction of the T2DM cohort assumed that an individual had already developed the disease prior to the study if the SUPREME-DM algorithm identified it at any point during the study period. We made this assumption for the following reasons. To take advantage of the consistent and high-quality records in ICD-10, we limited our study period to the time after Johns Hopkins’s transition to the ICD-10 system. This resulted in only 3 years of data on our cohort. Attempting to confirm T2DM prior to the inclusion in the study would have further reduced our sample size and statistical power. Nonetheless, the confirmed disease during the study period likely indicates the onset prior to the period, especially in our older study population whose average age (61.4) is significantly above the average age of T2DM diagnosis (49.9) in American adults.^37^ Second, our study could suffer from informative censoring since patients with social issues, especially those experiencing transportation hardship, had less access to hospital-based care and in turn were more prone to get lost to follow-up. This censoring would result in fewer observations and reduced power. This will bias the results towards the null but is unlikely to change our conclusion. Third, the SCCS method may not control time-varying confounding factors such as the neighborhood environment, which could affect patients’ HbA1c levels.^10^ We partially addressed this limitation by conducting a sensitivity analysis, in which we account for the increasing or decreasing trend of HbA1c independent of the SDoH exposure. The results from this sensitivity analysis, provided in Figure S1, show that our findings remain similar.

Despite the above limitations, our study provides an important piece of evidence to the literature studying how unmet social needs may compromise the health and well-being of patients with diabetes. Our study is the first of its kind to leverage the SCCS design to circumvent many of the confounding issues that are otherwise difficult to control in studying the impacts of SDoH on individuals’ health. Our findings continue to hold under sensitivity analyses and are consistent with the existing literature. In future work, the same analytic approach can be used on other populations to validate the current findings and potentially yield further insights. Future work may also incorporate SDoH data from administrative claims or those collected at the neighborhood level.^28^^,^^34^

## Conclusion

Confirming the absence of social needs is difficult from EHR records due to underreporting. This uncertainty in exposure makes it difficult to study the impact of SDoH on diabetes management. Unlike the traditional case-control designs, self-control designs circumvent this obstacle by using patients as their own controls. This study is one of the first attempts to apply this analytic strategy to assess the impact of social needs in association with diabetes management. We found that social issues overall are associated with poor diabetes management; transportation burden, especially, appears to be a potential barrier to glycemic control.

## Supplementary Material

ooae107_Supplementary_Data

## Data Availability

The data underlying this article were extracted from the electronic health record at the study site. The data are only accessible to researchers with an appropriate IRB approval. It cannot be shared publicly for the privacy of the patients in the study.
