# Supplementary material for: Assessing the impact of social determinants of health on diabetes severity and management
Source: JAMIA Open. 2024 Oct 25;7(4):ooae107. doi: 10.1093/jamiaopen/ooae107 (PMC11512144; doi:10.1093/jamiaopen/ooae107)

**Assessing the impact of SDoH on T2DM severity and management**

Xiyu Ding, MS^1^, Hadi Kharrazi, MD, PhD^1,2^, Akihiko Nishimura, PhD^3^

*^1^Biomedical Informatics and Data Science Division of General Internal Medicine,*

*Johns Hopkins University School of Medicine, Baltimore, MD, USA*

*^2^Department of Health Policy and Management,*

*Johns Hopkins Bloomberg School of Public Health, Baltimore, MD, USA*

*^3^Department of Biostatistics,*

*Johns Hopkins Bloomberg School of Public Health, Baltimore, MD, USA*

# **Supplementary**

Table S1. List of SDoH related ICD-10-CM codes

| **Domain** | **Description** | **ICD-10 Codes** |  |  |  |
| --- | --- | --- | --- | --- | --- |
| **Access to health care** | Difficulty of accessing healthcare service or facilities for some reasons | Z59.7, Z59.8, Z60.8, Z75.3, Z75.9, Z76.8, Z76.89, Z91.1, Z91.120, Z91.8, Z91.89 |  |  |  |
| **Food deficiency** | Problems or difficulty related to food deficiency, including nutritional deficiency, lack of adequate food and safe drinking water | E63.9, Z59.4, Z73.8, Z73.89 |  |  |  |
| **Housing** | Problems or difficulty related to housing | Y93.E, Y93.E6, Z59.0, Z59.1, Z59.1, Z59.8, Z77.0, Z77.011, Z77.1, Z77.120 |  |  |  |
| **Social connections isolation** | Problems related to social skills, living alone and family disruption issues. | R45.8, R45.89, Z60.2, Z60.4, Z60.8, Z63.0, Z63.5, Z63.8, Z63.9, Z65.9, Z73.4, Z91.8, Z91.89 |  |  |  |
| **Stress** | Physical and mental strain related to work, life events, and relaxation. | F43.2, F43.20, F43.9, Z56.3, Z56.6, Z63.7, Z63.79, Z63.8, Z73.2, Z73.3, Z73.8, Z73.89, Z91.8, Z91.89 |  |  |  |
| **Transportation** | Problems or difficulty related to transportation | Z59.8, Z76.8, Z76.89 |  |  |  |
| **Finance** | Problems or difficulty related to financial situation | Z59.7, Z59.8, Z59.5, Z59.6 |  |  |  |

Figure S1. Results of the CSCSS model with time adjustment

To explain differences across time (e.g., HbA1c levels might change as people get older), we introduce $t_{j}$, a time-dependent parameter, measuring the number of years between the $j_{\mathrm{th}}$ and the first HbA1c record. We assume the time effect is the same across patients. The model is therefore constructed as follows:

$$y_{\mathrm{ij}}|X_{ij}=\alpha_{i}+t_{j}+\beta^{T}X_{ij}+\epsilon_{ij}, \epsilon_{ij}\sim N(0,\sigma^{2})$$

Figure S1 summarizes the effects of SDoH factors on the HbA1c level estimated under the updated CSCCS model with the time-dependent variable. The results of such analyses are not much different from the model without the time adjustment. We found that experiencing social issues overall was associated with an increase of HbA1c level (%) by 0.065 (95% CI [0.010,0.120]) evaluated at window period +/- 45. More specifically, having transportation issues was associated with an increase of HbA1c by 0.100 (95% CI [0.009, 0.192]), 0.128 (95% CI [0.018, 0.238]), 0.120 (95% CI [0.005, 0.234]) evaluated at window period +/- 180, 60, 45. Food deficiency was associated with a decrease of HbA1c level by 0.306 (95% CI [-0.586, -0.026]) evaluated at window period +/- 30.


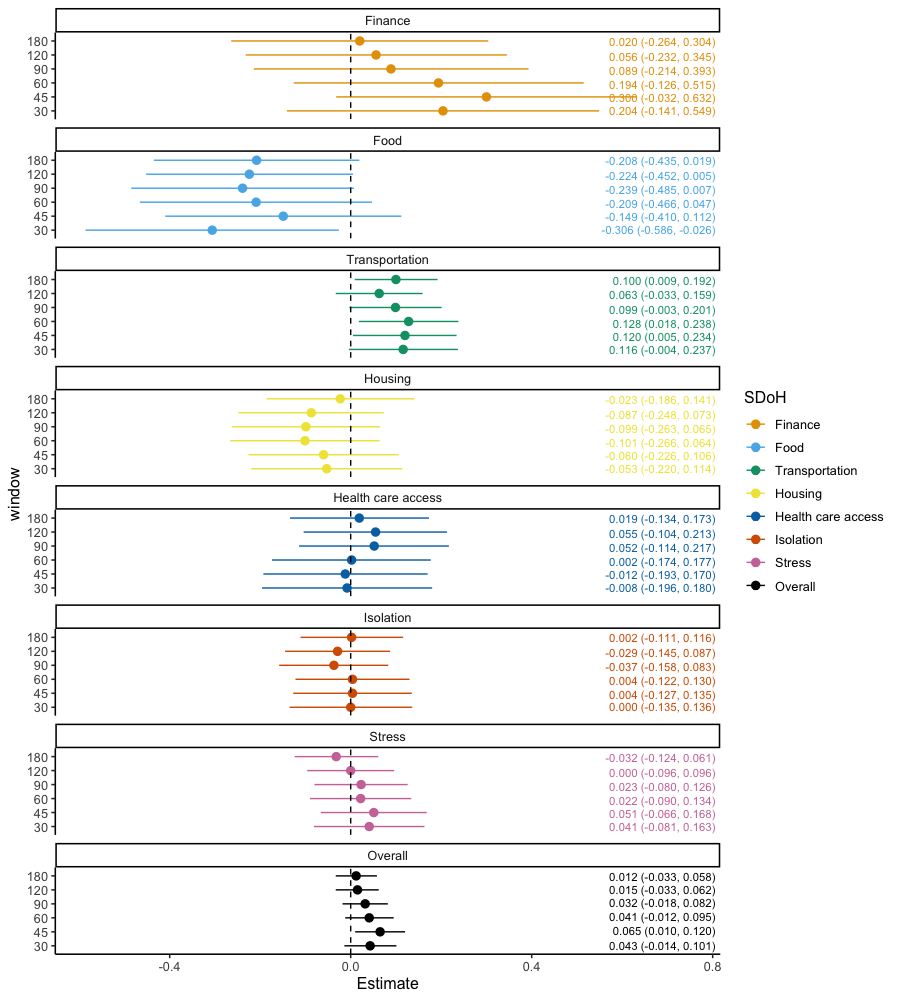

Supplement: ooae107_Supplementary_Data [file ooae107_supplementary_data.docx]
